# Iatrogenic insertion of impression mould into middle ear and mastoid and its retrieval after 9 years: a case report

**DOI:** 10.1186/1752-1947-1-3

**Published:** 2007-02-02

**Authors:** Mohammad Sohail Awan, Moghira Iqbal, Zakariya Imam Sardar

**Affiliations:** 1 Section of Otolaryngology Head and Neck Surgery, Department of Surgery, Aga Khan University Hospital, Karachi, Pakistan

## Abstract

The magnitude of hearing loss in Pakistan is enormous. One in twelve children of Pakistan suffers from some form of hearing impairment. Many of them are unable to afford surgical procedures and resort to the use of cheap hearing aids fitted by untrained individuals or people lacking the required expertise. This predisposes the patients to significant complications during a process that is otherwise considered safe.

We report the case of a child, where the process of making the mould for a hearing aid led to the perforation of the tympanic membrane and pouring of mould material into the middle ear, necessitating surgical intervention. During initial surgery it was thought that all mould had been removed from the middle ear but 9 years later this child underwent cochlear implantation at the same center and remaining part of ear mould was discovered from mastoid cavity.

## Introduction

The magnitude of hearing loss in Pakistan is enormous. One in twelve children of Pakistan suffers from some form of hearing impairment [1]. Many of them are unable to afford surgical procedures and resort to the use of cheap hearing aids fitted by untrained individuals or people lacking the required expertise. This predisposes the patients to significant complications during a process that is otherwise considered safe.

We report the case of a child, where the process of making the mould for a hearing aid led to the perforation of the tympanic membrane and pouring of mould material into the middle ear, necessitating surgical intervention. During initial surgery it was thought that all mould had been removed from the middle ear but 9 years later this child underwent cochlear implantation at the same center and remaining part of ear mould was discovered from mastoid cavity.

## Background

A 2 year old boy was brought to us by his parents for delayed speech development. There was no history of consanguineous marriage of parents. Pregnancy as described by mother was uneventful and there were no complications during delivery of this child. The birth weight of child was normal and there was no history of early infections like measles or meningitis. Clinical examination revealed intact tympanic membranes on both sides. Brainstem evoked response audiometry (BERA) showed bilateral severe peripheral hearing loss. As facilities of behavioral audiometery were not available at that time in our center and BERA was only done at two specific frequencies, the child was offered a hearing aid and was advised to follow up.

At 5 years of age the child required re-setting of his hearing aid apparatus. Silicone base ear mould material was injected into his ear to make impression for ear canal following which he developed bleeding from his right ear. This was treated conservatively until he presented to us 3 months after the incident with bleeding and purulent discharge from the same ear.

Outpatient examination revealed granulation tissue in the middle ear. A provisional diagnosis of traumatic perforation of tympanic membrane with suspicion of foreign body was made. Subsequently the granulation tissue was removed under general anesthesia. At the end of removal of granulation tissue, a bluish adherent material was seen to fill the whole middle ear, attic, aditus and Eustachian tube orifice. This was found to be the hearing aid impression material (silicone) that had entered the middle ear following the perforation of the tympanic membrane during the process of mould making. Hence by endaural approach this impression material was completely removed from the middle ear. However, the handle of malleus was totally embedded into the material and could not be preserved. Patch myringoplasty was performed. It was thought that was that all impression material had been removed from the ear. The child remained well post operatively and the drum healed nicely.

**Figure 1:**
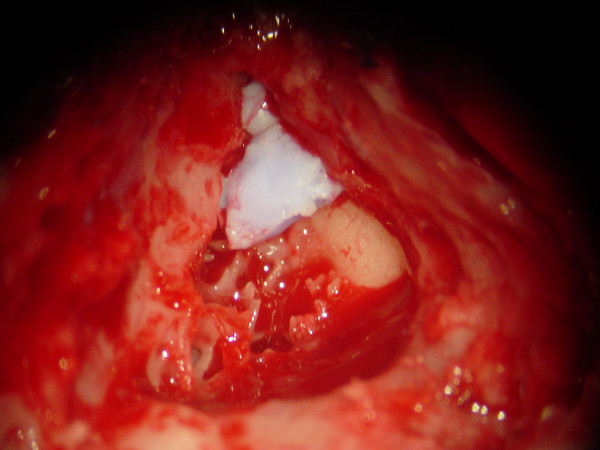
A soft, bluish foreign body seen to fill the mastoid antrum up to the attic area.

At the age of 14 years, the patient was listed as a candidate for cochlear implantation. M.R.I. scans before the surgery were unremarkable. As cortical mastoidectomy was being performed a soft, bluish foreign body was seen to fill the mastoid antrum up to the attic area (see Figure 1). This was part of the impression material that had entered the middle ear during the mould making process 9 years back and had gone all the way into the mastoid antrum – an area that had not been explored back then.

The material however did not elicit any tissue reaction and was not adherent to incus and facial nerve. It was removed once it was completely exposed. The cochlear implant was successfully inserted. The patient remained well in the postoperative period.

## Discussion

The making of ear mould for hearing aids is generally considered to be a safe process. However, there are a few reported cases of complications caused during mould making. One center from Netherlands reported accidental pouring of mould making material into the middle ear through a pre-existing perforation of the tympanic membrane, necessitating tympanotomy for its removal [2]. Another case is reported of iatrogenic perforation of the tympanic membrane by the mould material [3]. This case required surgical intervention for removal of material by employing mastoidectomy with facial recess approach to the middle ear. In this instance the hearing mechanism of the ear was compromised leading to further hearing impairment. One case report from USA and another from Poland also exemplify similar iatrogenic middle ear trauma resulting from ear impressions, and necessitating subsequent surgery [4,5].

Our case is also an example of iatrogenic perforation of the tympanic membrane and resultant pouring of the mould material into the middle ear cavity as well as mastoid. In this case we were able to remove the material completely from the middle ear, although the handle of malleus had to be sacrificed. However, mastoid remained an unsuspected site that harbored the material for 9 years before being incidentally found during cochlear implantation. Even a pre-operative M.R.I. scan failed to highlight the presence of the material in the mastoid.

Our case highlights some important points for consideration. Mould making by untrained hands can result in significant complications leading to further hearing impairment and disability. An appropriate material should be chosen for the mould and care should be taken not to push it in the ear canal with too much pressure. The ear canal should not be sealed off by the piston so that if the pressure rises in the ear canal, the material has space from which to flow out instead of causing trauma to the tympanic membrane [2].

Furthermore there needs to be a close liaison between the Otolaryngologist and the audiologist/Vendor of the hearing aid and any incident of such nature warrants immediate referral to a tertiary care center for further management. We also suggest registration of all hearing aid centers with a central licensure authority to ensure that they meet a minimum standard in expertise and equipment.

We conclude that the ear mould injection for impression of the ear canal for hearing aids can result in disastrous consequences when performed by poorly trained individuals. Such cases are likely to be more frequent, but remain highly under reported.

## Competing interests

The author(s) declare that they have no competing interests.

## Authors' contributions

MSI conceived of the case, and participated in its formatting and coordination and helped to draft the manuscript. ZIS helped in drafting the report and literature review. MI reviewed the case, helped in drafting the report. All authors read and approved the final manuscript.
